# Research progress on generation, detection and control of hazards in baked foods during thermal processing

**DOI:** 10.1016/j.fochx.2025.103252

**Published:** 2025-11-07

**Authors:** Qi Zhang, Pei Liu, Yanhong Ma, Yuhua Diao, Ying Gu, Xuejing Fan

**Affiliations:** a Faculty of Food Science and Engineering, Kunming University of Science and Technology, Kunming, 650500, China; b Kunming Institute for Food and Drug Control, Kunming, Yunnan, 650032, China

**Keywords:** Baked foods, Hazards, Generation mechanisms, Detection methods, Control strategies

## Abstract

Thermal processing techniques, such as baking, play a crucial role in imparting desired sensory characteristics to food. However, these processes simultaneously lead to the formation of various *thermal processing hazards (TPHs)*, including *acrylamide (AA)*, *5-hydroxymethylfurfural (5-HMF)*, *furan*, *advanced glycation end products (AGEs)*, *heterocyclic amines (HCAs)*, and *imidazoles (MI)*. While extensive research has focused on the formation and control of individual categories of hazards, studies investigating the synergistic effects and coordinated mitigation of multiple *TPHs* remain scarce. This review comprehensively examines the mechanisms of TPH generation, detection methodologies, and control strategies. Importantly, it proposes viable approaches for the synergistic mitigation of multiple hazards, offering key insights to support researchers and industry practitioners in advancing food safety and quality.

## Highlights

• Baked foods exhibit diverse thermal processing hazards.
• Different hazards have similar reaction pathways and intermediate products.
• Carbonyl compounds are critical to the formation of hazards.
• Propose synergistic control methods to enhance the safety of baked foods.

## 1 Introduction

Thermal processing is a critical step in food production, widely employed to modify the physicochemical properties of ingredients, such as protein denaturation, starch gelatinization, and pectin degradation, inactivate spoilage microorganisms and enzymes, and enhance flavor via the Maillard reaction. Depending on processing temperature and the thermal transfer medium, this essential step is broadly categorized into dry and wet thermal processing. Dry thermal processing, including roasting, frying, baking, and smoking (Oke, Idowu, Sobukola, Adeyeye, & Akinsola, 2018; Baldino, Lupi, & Gabriele, 2023; Šimko, 2002), generally occurs at temperatures above 100 °C, with heat transfer achieved through thermal air circulation or direct contact with heated surfaces (Sun, Gong, Li & Xiong, 2014). Common processing methods include boiling, braising, steaming, pasteurization, and ultra-high temperature sterilization (Xu & Chang, 2008; Ramesh, 2020; Ao et al., 2022). In addition to these conventional methods, novel thermal technologies are emerging to improve processing efficiency and product quality. Superheated steam (SHS) utilizes high-temperature steam in an oxygen-free environment, creating ideal conditions for inhibiting lipid oxidation and reducing the formation of hazardous compounds like *acrylamide*. This technology not only efficiently reduces microbial contamination on food surfaces through its high heat transfer coefficient but also better preserves product quality by minimizing nutrient loss and controlling protein denaturation with functional characteristics, as demonstrated in its application to various food matrices including cereals and pollen products (Bi, Zhang, Qiao, & Xu, 2017; Fang et al., 2023). Ohmic heating generates heat instantaneously and uniformly throughout the food by applying an electric current, minimizing thermal degradation and preserving the quality of heat-sensitive compounds. Similarly, microwave-assisted processing employs electromagnetic waves for rapid volumetric heating, significantly shortening process time and improving nutrient retention compared to conventional methods (Jaeger et al., 2016; Li et al., 2023). Bakery products such as bread, cakes, biscuits, and pastries are major dry-processed foods and daily dietary staples worldwide (Ghnimi, Ennouri, Chèné, & Karoui, 2023). However, their reliance on high-temperature dry thermal processing makes them especially prone to *thermal processing hazards (TPHs)* (Capuano & Fogliano, 2011). Thermal processing enhances flavors and aromas but also produces *TPHs* such *acrylamide (AA)*, *5-hydroxymethylfurfural (5-HMF)*, *furan*, *advanced glycation end products (AGEs)*, *heterocyclic amines (HCAs)*, and *imidazoles (MI)*. (Choudhary et al., 2021; Hee, Liang, Zhang, & Fang, 2023; Miśkiewicz, Rosicka-Kaczmarek, & Nebesny, 2020; Zhang et al., 2024). The main challenge is mitigating *TPHs* while retaining the sensory quality of baked goods, despite available reduction methods (Barrios-Rodríguez, Gutiérrez-Guzmán, Pedreschi, & Mariotti-Celis, 2022). Growing evidence of their health risks has further raised public concern over food safety (Li et al., 2023; Guo et al., 2025; Wang, Li, Liu, & Jiang, 2025; Liu et al., 2025).

However, while the scientific literature is rich with reviews on individual *TPHs*, there is a conspicuous lack of comprehensive analyses dedicated to the collaborative control of multiple hazards, particularly within the context of bakery products. This review aims to bridge this gap. Its primary distinction lies in shifting the focus from a single-hazard perspective to an integrated one, specifically for baked goods. We systematically summarize the intersecting formation pathways of co-existing *TPHs* in these products and discuss control strategies with the potential for simultaneous mitigation of multiple hazards. By synthesizing this knowledge, our objective is to clarify the challenges and opportunities in multi-hazard control, thereby providing a stronger theoretical foundation for researchers and industry stakeholders.

### 1.1 Literature search strategy

**Fig. 1:**
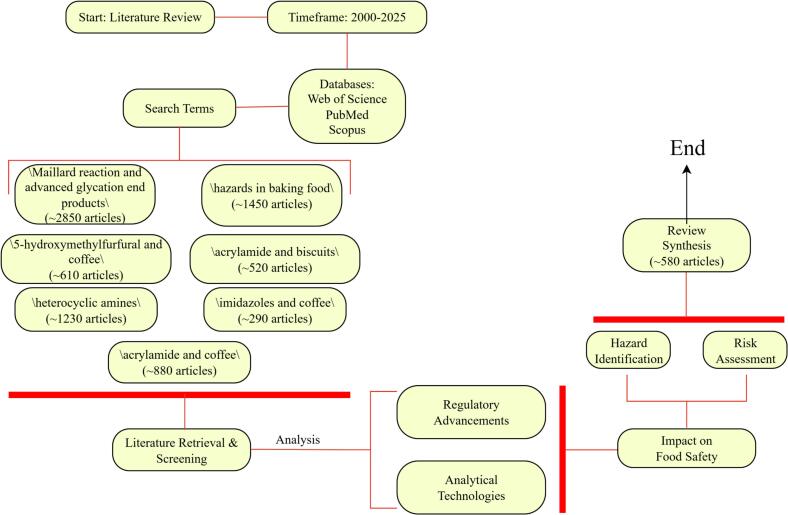
Flowchart of literature Retrieval.

This review synthesizes findings from relevant scientific articles published between 2000 and 2025, a timeframe selected to capture pivotal advancements in food safety regulations and analytical technologies that fundamentally reshaped hazard identification and risk assessment in thermally processed foods. Literature was sourced from the Web of Science, PubMed and Scopus databases. Search terms included: “hazards in food baking”, “*acrylamide* and coffee”, “*acrylamide* and biscuits”, “*5-hydroxymethylfurfural* and coffee”, “*heterocyclic amines*”, “maillard reaction and *advanced glycation end products*”, and “*imidazoles* and coffee”. The specific retrieval process is shown in Fig. 1.

## 2 *TPHs* formation mechanisms

### 2.1 *AA* formation mechanism

*AA* (C_3_H_5_NO), a compound capable of damaging human nerves, is primarily found in carbohydrate-rich foods such as coffee, grains, and potatoes (Li, Kahlon, Wang, & Friedman, 2021). Notably, the WHO's International Agency for Research on Cancer (IARC) has classified *AA* as a Group 2 A probable human carcinogen (Zuckerman, 1995).

Tareke, Rydberg, Karlsson, Eriksson, and Törnqvist (2002) first investigated the formation mechanism of *AA*, a thermally processed hazard, in foods and reported that higher carbohydrate content and elevated processing temperatures increase both the likelihood and levels of *AA* formation. While *AA* formation begins at temperatures around 120 °C, the optimal range for its generation is approximately 160–180 °C (Karami, Akbari-Adergani, Jahed Khaniki, Shariatifar, & Sadighara, 2022; Khan et al., 2017), which explains its frequent presence in baked and fried foods, whereas microwave- or boil-processed foods typically contain lower levels (Abt et al., 2019; Kocadağlı & Gökmen, 2022; Nematollahi et al., 2020). Coffee is a notable thermally treated food with high *AA* content, averaging 522 μg/kg, with instant coffee exhibiting significantly higher levels (700–1200 μg/kg) than roasted coffee (200–400 μg/kg), primarily due to additional processing steps. During spray-drying of coffee extract, prolonged exposure to 150–200 °C promotes *AA* formation via the Maillard reaction between asparagine and reducing sugars. The inclusion of vegetable fat powder (10–15% *w*/w) as an anti-caking agent contributes further, as its unsaturated fatty acids, such as linoleic acid, undergo oxidation during drying or storage to generate reactive carbonyls (e.g., acrolein), which participate in alternative *AA* formation pathways either by reacting with asparagine or fragmenting to form acrylic acid (Guenther, Anklam, Wenzl, & Stadler, 2007; Holkar, Jadhav, & Pinjari, 2019; Schouten et al., 2021; Vezzulli, Triachini, Mulazzi, Lambri, & Bertuzzi, 2022). The European Commission has established regulatory reference levels (RLs) of *AA* in coffee products, specifying 400 μg/kg for roasted coffee and 850 μg/kg for instant coffee preparations. *AA* formation dynamics in coffee are governed by complex interactions between intrinsic and processing-related parameters. Intrinsic determinants primarily involve post-harvest handling variables, particularly storage conditions (including seasonal variations in temperature and humidity) that influence precursor compound availability. Extrinsic modulators encompass thermal processing parameters (time-temperature profiles during roasting) and formulation strategies involving exogenous additives that may alter the Maillard reaction pathways (Claeys, De Vleeschouwer, & Hendrickx, 2005). Meanwhile, the reaction between reducing sugars and asparagine is governed by water activity (optimal 0.4–0.6), which enhances reactant mobility and reaction kinetics, and by pH, which affects the formation of key intermediates, collectively modulating reaction rates and *AA* yield (Claeys et al., 2005). In general, *AA* was formed by the following mechanisms.

#### 2.1.1 Reaction of reducing sugars with asparagine

The formation of *acrylamide (AA)* primarily originates from the reaction between asparagine (Asn) and carbonyl precursors, most notably reducing sugars, during the initial phase of the Maillard reaction. This reaction pathway is profoundly influenced by extrinsic factors such as water activity, pH, and thermal input (Claeys et al., 2005). The mechanism is initiated by the nucleophilic attack of the α-amino group of asparagine on the carbonyl group of a reducing sugar, leading to the formation of a reversible N-glycosylasparagine adduct. This adduct swiftly dehydrates to form a key transient intermediate, the Schiff base. It can then proceed through two principal competitive pathways. It may undergo direct deamination and decarboxylation to yield a decarboxylated Schiff base, which can subsequently hydrolyze to form *AA*. Alternatively, and more prevalently in low-moistry systems like baked goods, the Schiff base cyclizes and rearranges to form the more stable Amadori Rearrangement Product (ARP). The degradation of this ARP under elevated temperatures serves as a critical branching point, generating a diverse and complex pool of highly reactive α-dicarbonyl compounds (e.g., glyoxal, methylglyoxal, 3-deoxyglucosone) and other intermediates like oxazolidin-5-one. It is critical to note that parallel to the ARP pathway, the direct thermal degradation of sugars (e.g., fructose and glucose) also contributes significantly to this dicarbonyl pool. The final and decisive step involves the condensation of these generated α-dicarbonyl compounds with free asparagine. This reaction proceeds via Strecker degradation, a process characterized by the oxidative deamination and decarboxylation of the amino acid, which directly furnishes the *AA* molecule (Nguyen, Peters, & Van Boekel, 2016; Rannou, Laroque, Renault, Prost, & Sérot, 2016). This elaborated network underscores a key concept: while the diversity and abundance of reactive carbonyl species are crucial drivers of *AA* yields, the availability of free asparagine remains the primary limiting factor in most cereal-based baked products, where reducing sugars are often present in substantial excess (Barrios-Rodríguez et al., 2022).

#### 2.1.2 Other approaches

Baskar and Aiswarya, (2018) showed that acrolein and acrylic acid are key precursors of *AA* in the non-asparagine pathway. Free asparagine reacts with acrolein, whose carbonyl group promotes large-scale *AA* formation (Lingnert et al., 2002). Acrolein arises from glycerol dehydration during glycerol trioleate degradation or from acetaldehyde oxidation (Chen et al., 2022). Acrylic acid, formed by acrolein oxidation, can also react with ammonia to yield *AA* (Maan et al., 2022). Moreover, monosaccharide decomposition may generate acrylic acid under specific conditions (Yasuhara, Tanaka, Hengel, & Shibamoto, 2003). Subsequently, these small molecules react to form *AA*, providing an alternate pathway for *AA* formation. Friedman (2003) & Lingnert et al. (2002) further showed that acrolein obtained from amino acid catabolism, lipid oxidation and carbohydrate decomposition simultaneously affects *AA* formation in the whole Maillard reaction. Thermally labile compounds such as aspartic acid, carnosine, and β-alanine also contribute to *AA* generation, as acrylic acid formed during their degradation reacts with amino acids (Stadler et al., 2003). Additionally, *5-HMF*, produced during food roasting, particularly in coffee, forms prior to *AA* and significantly promotes its formation with increased roasting intensity. The carbonyl group in *5-HMF* enables rapid reaction with asparagine during the Maillard reaction. Cai et al. (2014) proposed three mechanisms by which chlorogenic acid enhances *AA* formation: by increasing *5-HMF* production during roasting, lowering the activation energy for converting 3-aminopropionamide to *AA* (from 173.2 kJ/mol to 136.6 kJ/mol), and maintaining a high redox potential during the Maillard reaction, which protects *AA* from degradation.

### 2.2 *5-HMF* formation mechanisms

*5-HMF* is a furan derivative formed through the thermal decomposition of sugars and is widely utilized in the food industry as a quality marker and adulteration indicator for products such as coffee, juices, milk, sauces, honey, and cereals (Capuano & Fogliano, 2011). International regulatory standards set critical limits for *5-HMF*, with the WHO and EU establishing a maximum threshold of 40–80 mg/kg in honey, while EU regulations restrict concentrations to 20 mg/kg in infant fruit juices and 50 mg/kg in other juices (Ye et al., 2022). Additionally, *5-HMF* contributes to the aroma of roasted coffee. Although the International Agency for Research on Cancer (IARC) does not currently classify *5-HMF* as a carcinogen, its metabolites have been extensively studied for potential genotoxic and carcinogenic effects. In vitro studies indicate that the metabolite 5-sulfoxymethylfurfural (SMF) induces DNA adducts in renal cells at cytotoxic doses (>250 mg/kg in mice) and causes neurotoxicity via oxidative stress in neuronal cell lines (PC12/HT22) at 100 μM concentrations (Jiang, Zhong, Wang, & Zhang, 2022). Furthermore, *5-HMF* has been shown to induce mutations, potentially promote cancer progression, deplete cellular glutathione levels, and trigger allergic responses (Xie, Wang, Zhao, & Zhou, 2023). The formation of *5-HMF* predominantly occurs through the Maillard reaction and caramelization, with the Maillard reaction considered the principal pathway.

#### 2.2.1 Maillard and caramelization reactions

The formation of *5-HMF* occurs via two primary pathways. The first involves the Maillard reaction, in which amino acids react with glucose or fructose to form Schiff bases that undergo dehydration through enolization, yielding 3-deoxyglucosone (3-DG) (Zhang et al., 2019). Immediately afterwards, 3-deoxyglucosone (3-DG), formed via 1,2-enolization of Amadori rearrangement products, undergoes acid-catalyzed (H^+^) β-elimination to yield 3,4-dideoxyglucosone (3,4-DG) through a two-step dehydration mechanism, *5-HMF* is finally formed by cyclization. Notably, the formation of *5-HMF* was directly related to the concentration of 1,2-enaminol, and the 3-deoxyglucone produced by 1,2-enaminol was the key intermediate in the dehydration of 3,4-dideoxyglucone to produce *5-HMF* (Capuano & Fogliano, 2011; Perez, Mukherjee, & Dumont, 2019). Another method was to directly hydrolyze sugar under acidic conditions and generate *5-HMF* through caramelization reaction (Hamzalıoğlu & Gökmen, 2018). When thermal-treated under acidic conditions, fructose was degraded to produce the fructofuranosyl cations. This reaction was known as caramelization and usually occured at temperatures in the range of 150 to 200 °C. Below 150 °C, the thermal energy is insufficient to overcome the activation energy required for the cleavage of glycosidic bonds and the formation of fructofuranyl cations. Generally speaking, the processing temperature of baked goods such as cookies, coffee beans and hazelnuts is usually within this temperature range (Capuano & Fogliano, 2011; Goncuoglu Tas & Gokmen, 2017; Perez et al., 2019).

Notably, the efficiency of *5-HMF* production is influenced by multiple factors, including cation type, amino acid concentration, water activity, carbohydrate type, temperature, and pH. Among these, high fructose content and the presence of chloride and calcium ions favor *5-HMF* production. High water activity, however, inhibits carbohydrate dehydration, thereby inhibiting *5-HMF* formation. Fructose and glucose are known to be interconvertible, but the efficiencies with which they form *5-HMF* differ significantly. Fructose is much more reactive and selective for *5-HMF* formation than glucose, as glucose dehydration produces numerous byproducts, including cross-aldol condensation or polymerization. Therefore, increased isomerization efficiency translates to higher *5-HMF* yields (Anese & Suman, 2013).

### 2.3 *Furan* formation mechanisms

*Furan* formation has been demonstrated from various precursors and intermediates. These primarily include amino acids, carbohydrates, polyunsaturated fatty acids (PUFAs), ascorbic acid, and carotenoids. Consequently, *furan*s are widely present in diverse food products (Crews & Castle, 2007). The primary pathways contributing to *furan* formation during food processing include the thermal degradation of carbohydrates and amino acids, the Maillard reaction, and the thermal oxidation of ascorbic acid, carotenoids, and polyenoic fatty acids, as summarized in Table 1 (Owczarek-Fendor et al., 2011). These chemical reactions collectively contribute to *furan* as a thermal processing hazard (Limacher, Kerler, Conde-Petit, & Blank, 2007; Limacher, Kerler, Davidek, Schmalzried, & Blank, 2008; Owczarek-Fendor et al., 2011; Perez Locas & Yaylayan, 2004). Many studies have focused on lipid oxidation, with model systems demonstrating that the intermediate 4-hydroxy-2-butenal oxidizes to dihydro-2-furanol and subsequently forms *furan* through dehydration (Kettlitz et al., 2019). Liu et al. (2025) reported that linolenic acid, a polyunsaturated fatty acid, acts as a *furan* precursor via its oxidation to hydroperoxides, which then degrade into conjugated dienes and aldehydes capable of cyclization and dehydration to form *furan*. Additionally, factors such as food composition, processing time and temperature, and the presence of metal ions significantly influence *furan* production in food (Kettlitz et al., 2019; Ramírez et al., 2022). Based on simple model systems, researchers have proposed several pathways for *furan* formation involving single or multiple precursors, including: (a) thermal degradation and rearrangement during caramelization, (b) thermal degradation of amino acids, (c) Maillard reactions between amino acids and reducing sugars, (d) thermal oxidation of ascorbic acid, and (e) oxidation of PUFAs and carotenoids (Fan, 2005; Limacher et al., 2007; Limacher et al., 2008; Perez Locas & Yaylayan, 2004).

**Table 1:** Furan formation pathways in thermal processing.

**Generation approach**	**Core chemical mechanism**	**Main precursor substances**
Thermal degradation in caramelization	Sugar dehydration & cyclization	Reducing sugars
Direct thermal degradation of amino acids	Strecker degradation/decarboxylation	Serine, cysteine, threonine
Maillard reaction	Sugar-amine condensation→1-deoxyketose→furanals	Reducing sugars + amino acids
Thermal oxidation of ascorbic acid	C6-hydroxyl cleavage→2-deoxyaldose→cyclization	Ascorbic acid
Oxidative cleavage of PUFAs/carotenoids	Lipid peroxidation→4-hydroperoxy-2-alkenals→cyclization	Linoleic acid (ω-6); α-linolenic acid (ω-3); β-carotene

### 2.4 *AGEs* formation mechanisms

*AGEs* formed during thermal processing can be classified according to their chemical structures and precursor pathways, including lysine-derived modifications such as carboxymethyl lysine (CML), carboxyethyl lysine (CEL), and pyrraline; arginine-derived modifications such as methylglyoxal-derived hydroimidazolone-1 (MG-H1) and glyoxal-derived hydroimidazolone-1 (G-H1); and cross-linking structures including glyoxal-lysine dimer (GOLD), methylglyoxal-lysine dimer (MOLD), and pentosidine. Studies have found that CML, CEL, MG-H_1_ and G-H_1_ are closely related to diabetic complications and cardiovascular diseases (Dong et al., 2023). In addition to Maillard reaction, oxidation of lipids and reducing sugars can also produce *AGEs* (Lu et al., 2022). These substances were produced both in organisms and foods. *AGEs* found in baked products were mainly CML and CEL, both endogenously in organisms and exogenously in foods. In baked products, *AGEs* are primarily CML and CEL, which lack fluorescence and protein cross-linking structures (Van Nguyen, 2006; Zhang, Wang, & Fu, 2020). Significant variations in CML and CEL levels have been observed across different food categories, reflecting differences in composition and processing conditions, with bread and cookies exhibiting the highest CML content of 178.36 mg/kg.

First, the amino group of a protein, peptide, or amino acid reacts with the carbonyl group of a reducing sugar to form a Schiff base. The Schiff base then rearranges to form an Amadori rearrangement product, which in turn generates highly reactive α-dicarbonyl compounds such as glyoxal, methylglyoxal, and 3-deoxyglucose ketone (Liu et al., 2017). Finally, the α-dicarbonyl compound reacts with lysine or arginine residues to form various *AGEs*. It is noteworthy that while the classic Maillard reaction typically generates *AGEs* via stable Amadori rearrangement products, the Amadori products can also be oxidatively cleaved, bypassing subsequent intermediate stages and directly generating reactive carbonyl groups or *AGEs*. Beyond these pathways, spontaneous oxidation of reducing sugars, oxidative cleavage of Schiff bases, and lipid peroxidation also contribute to the formation of α-dicarbonyl compounds, which in turn participate in AGE formation (Zhang et al., 2020).

### 2.5 *HCAs* formation mechanisms

*HCAs* are foodborne carcinogens primarily present in protein-rich foods, and their formation via the Maillard reaction is influenced by factors such as the type of meat, thermal processing temperature and duration, and moisture content (Cheng et al., 2006). Additionally, the presence of fat can affect heat and mass transfer during thermal processing, thereby influencing HCA formation to a certain extent (Crews & Castle, 2007).

*HCAs* are generally classified into thermal *HCAs*, which are typically formed at lower temperatures ranging from approximately 100–300 °C (most commonly 140–180 °C during typical cooking), and pyrolytic *HCAs*, which are produced at higher temperatures, generally above 300 °C, often through direct pyrolysis. Thermal *HCAs* are polar compounds formed via the Maillard reaction pathway, involving creatine, amino acids, and carbohydrates, and progressing through intermediates such as the Amadori rearrangement and Strecker degradation. In contrast, pyrolytic *HCAs* are nonpolar compounds primarily generated through the high-temperature pyrolysis of amino acids or proteins, with little to no involvement of creatine (Crews & Castle, 2007).

**Fig. 2:**
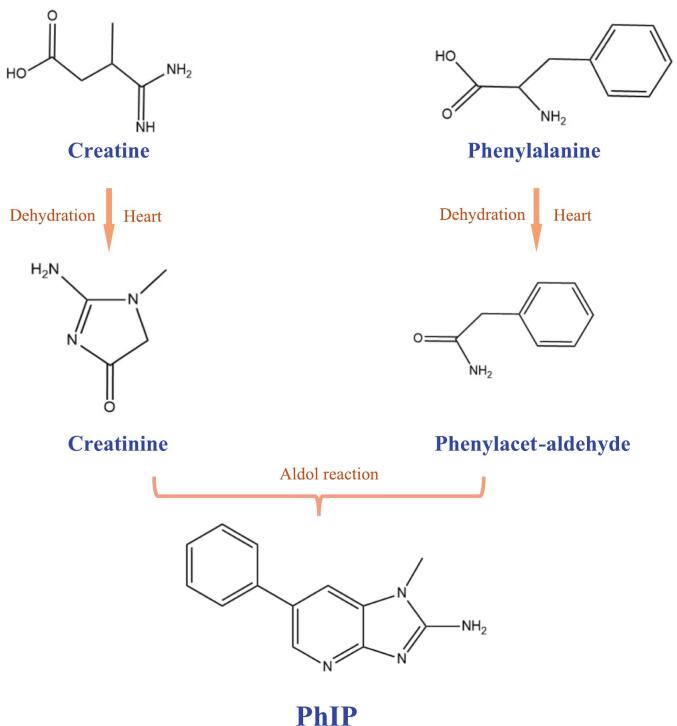
Generation mechanism of PhIP.

Among the hazards associated with *HCAs*, the imidazopyridine-type amine 2-amino-1-methyl-6-phenylimidazopyridine (PhIP) has been extensively studied (Crews & Castle, 2007). During thermal processing, such as baking, phenylalanine participates in Strecker degradation with creatine to generate phenylacetaldehyde and creatinine. Phenylacetaldehyde then undergoes an aldol-type condensation with creatinine, followed by dehydration and cyclization steps, ultimately forming PhIP. The detailed mechanism of PhIP formation is illustrated in Fig. 2.

### 2.6 Mechanisms of *imidazoles (MI)* generation

*MI* in bakery products, primarily *4-methylimidazole (4-MI)* and *2-methylimidazole (2-MI)*, are nitrogen-containing heterocyclic compounds formed under alkaline conditions (pH > 7) and high temperatures. Under these conditions, dicarbonyl compounds generated during the Maillard reaction act as precursors, reacting with ammonia to form either *2-MI* or *4-MI*. Research shows that hydroxyl (HO•) and carbon-centered free radicals are key to this process (Hong, Park, & Lee, 2020; Xue et al., 2023). Two main pathways are involved: (1) a dicarbonyl compound reacts with ammonia to form one intermediate, while a carbonyl compound (e.g., aldehyde) reacts with ammonia to form another; their condensation produces *2-MI* or *4-MI*. (2) a dicarbonyl compound reacts with ammonia to form a diammonium species, which then reacts with a carbonyl compound to yield *2-MI* or *4-MI* (Moon & Shibamoto, 2011; Wu, Huang, Kong, & Yu, 2015).

### 2.7 Relevance of multi-hazard generation mechanisms

**Fig. 3:**
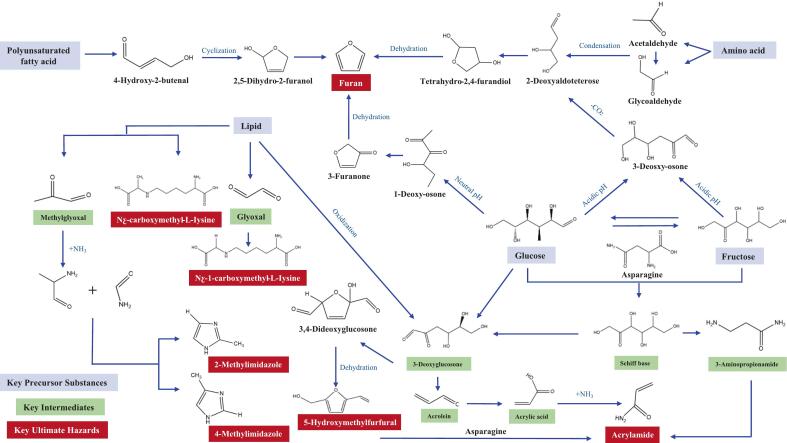
Formation mechanism and correlation of different hazards.

There are significant mechanism similarities in the reaction pathways and key intermediates for the formation of *TPHs*: *AA*, *5-HMF*, *AGEs*, *MI*, and *furan* derivatives. These compounds often share common precursors, including reducing sugars from the Maillard reaction and caramelization (e.g., glucose, fructose), amino acids (with asparagine limiting *AA* formation and lysine and arginine being critical for *AGEs*), and lipid substances, which can influence the formation of *furan* and acrolein. Central to these reactions is the generation of highly reactive carbonyl intermediates, particularly short-chain α-dicarbonyl compounds (α-DCs) such as glyoxal (GO), methylglyoxal (MGO), and 3-deoxyglucosone (3-DG), which serve as key branching points in complex reactions with electrophilic intermediates like acrolein and small fatty aldehydes (e.g., formaldehyde and acetaldehyde) (Barrios-Rodríguez et al., 2022). However, the intricate networks formed by the Maillard reaction, Strecker degradation, lipid oxidation, and caramelization present significant analytical challenges. By effectively inhibiting the formation of these core intermediates, particularly α-DCs, through strategies such as pH optimization, addition of inhibitors, or reduction of processing temperature and time, or by eliminating already formed intermediates, the production of *TPHs* dependent on these intermediates can be simultaneously minimized. The mechanisms of formation and interrelationships among these various hazards are illustrated in Fig. 3.

## 3 Detection methods and control technologies for common hazards in thermal treatment processes

**Fig. 4:**
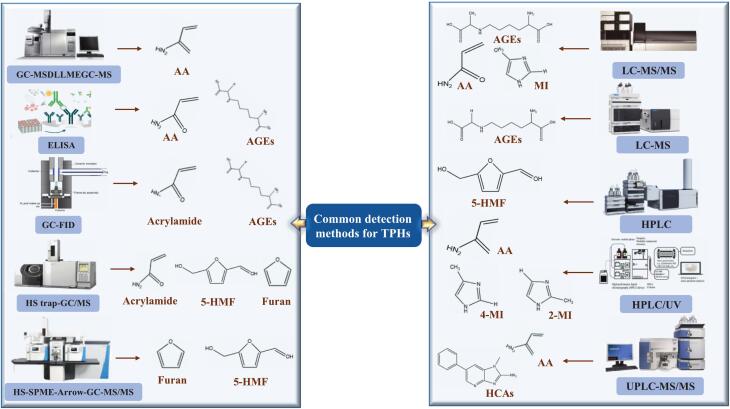
Detection methods of hazards in baked foods.

The detection of hazards in bakery products currently relies on a diverse array of techniques, each with specific advants and limitations suited to different applications. Enzyme-linked immunosorbent assays (ELISA) provide ease of use, high throughput, and relatively low cost, making them valuable for large-scale screening of specific allergens or microbial toxins, such as gluten or aflatoxins; however, their performance may be affected by cross-reactivity or matrix interference, potentially leading to false results. Aptamers and biosensors offer high specificity and sensitivity, often enabling rapid, on-site, or point-of-care detection of contaminants such as pathogens (e.g., Salmonella) or small molecule toxins. However, their wider application may be hindered by the complexity of probe design/manufacturing and sensitivity to operating conditions such as temperature and pH. Fluorescent probes are powerful tools for real-time imaging and spatial mapping of specific analytes in complex matrices, but challenges remain, such as photobleaching and a potential lack of absolute specificity for complex targets (Chen et al., 2022; Gómez-Ojeda et al., 2018; Khoshbin, Moeenfard, Abnous, & Taghdisi, 2023; Liang et al., 2022). Therefore, to accurately identify and quantify common thermal processing hazards (e.g., *AA*s, *furan*s, *5-HMF*, or *AGEs*), most laboratories prioritize more robust and specific chromatographic techniques coupled to mass spectrometry. These techniques include gas chromatography-flame ionization detection or mass spectrometry (GC-FID/MS), liquid chromatography-ultraviolet detection and mass spectrometry (LC-UV/MS), and ultra-high performance liquid chromatography (UPLC-MS), often coupled with mass spectrometry. These methods offer excellent sensitivity, selectivity, and the ability to analyze multiple analytes simultaneously. However, their high instrumentation and operating costs, coupled with the requirement for significant technical expertise, often make them unsuitable for routine monitoring in resource-limited settings, such as small bakeries. Emerging technologies are gaining increasing attention from researchers, driven by the need for more convenient, faster, and more reliable solutions. These technologies include novel biosensing platforms that utilize nanomaterials (e.g., graphene, quantum dots) or synthetic biology elements to enhance signal transduction, and advanced spectroscopic techniques such as surface-enhanced Raman spectroscopy (SERS) combined with chemometrics, which offer promising avenues for non-destructive, label-free, and potentially high-throughput screening of bakery product safety (Bachir, Haddarah, Sepulcre, & Pujola, 2023; Hu et al., 2022; Rafiei Jam, Nezhadali, & Kaykhaii, 2022; Verma & Yadav, 2022). Furthermore, the integration of machine learning (ML) with spectroscopic methods is forging a new paradigm for food hazard analysis. By employing advanced algorithms—including support vector machines and deep learning networks—to decode complex spectral data, ML-assisted spectroscopy significantly enhances detection sensitivity, accuracy, and broad-spectrum capability (He, Huang, & Wang, 2024). This approach is particularly effective for the simultaneous identification of multiple co-existing hazards, even in complex food matrices like bakery products, by extracting subtle, non-linear patterns that conventional methods miss (Ding, Xie, Yu, Cui, & Wilson, 2025). The principles and data processing workflows honed in general food component analysis is now being successfully migrated to the specific challenge of hazard detection, enabling more robust and reliable quantification (Li et al., 2024). This not only improves predictive accuracy but also facilitates rapid, non-destructive screening. Concurrently, progress in miniaturized sensor arrays, which utilize nanomaterials as receptors to generate distinct response fingerprints, is pivotal (Li et al., 2024). When coupled with lightweight ML models deployed on portable devices, these advances are effectively translating laboratory-grade accuracy into field-deployable tools. This synergy enables real-time, on-site hazard monitoring at critical points along the supply chain, dramatically improving portability and accessibility (Ding et al., 2025; He et al., 2024). The most commonly used detection methods are summarized in Fig. 4.

Mitigation of hazardous compounds in bakery products mainly relies on limiting precursor availability, optimizing processing, and adding exogenous agents (Zhang et al., 2024). Precursor type and concentration, particularly amino acids and sugars, strongly affect final hazard levels (Miśkiewicz et al., 2020). The formation of many hazardous compounds, including *AA*, *5-HMF*, and *AGEs*, is highly dependent on baking parameters, with their concentrations generally increasing at higher temperatures and longer baking times (Zhang et al., 2024). Although extended roasting may reduce certain hazards, it often severely compromises sensory quality and is therefore not a practical control strategy. The use of exogenous additives has emerged as a widely studied and effective approach to hazard reduction, with commonly applied compounds including amino acids, organic acids, cations, polyphenols, flavonoids, and hydrocolloids (Zhang et al., 2024).

**Fig. 5:**
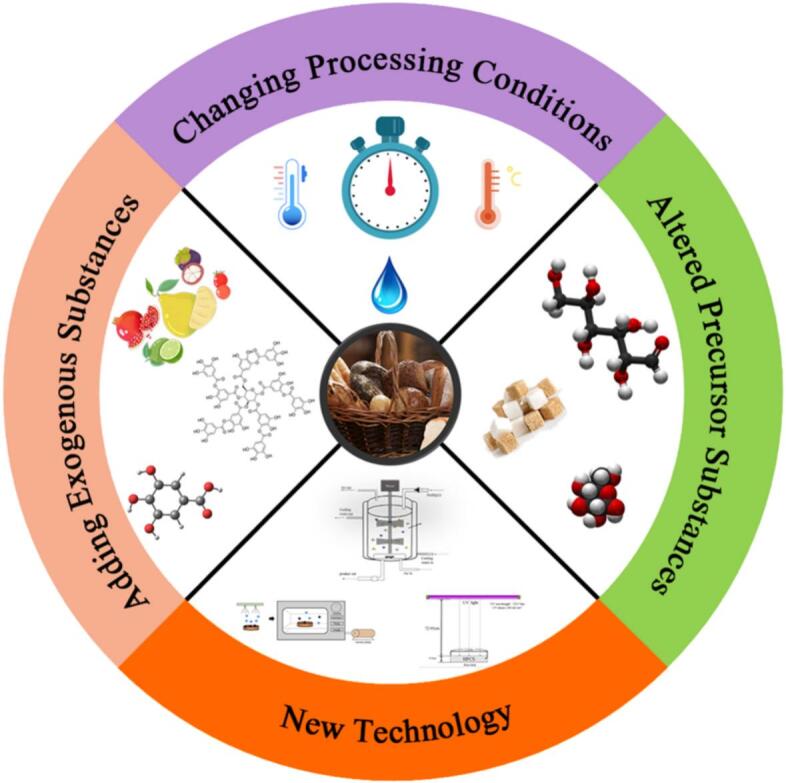
Control strategies for hazards in baked foods.

Bakery products require a certain degree of thermal treatment, which is the primary stage during which hazardous compounds accumulate. The rapid development of novel food processing technologies, including vacuum heating, microwave heating, and radio frequency heating, offers alternative heat treatment methods for bakery products. When combined with traditional heating methods, these technologies can effectively reduce the formation of hazardous compounds. A summary of control strategies for common hazards during thermal processing is illustrated in Fig. 5.

### 3.1 *AA* detection methods and control methods and technologies

#### 3.1.1 Test methods for *AA*

Commonly used methods for *AA* detection include LC-MS/MS, LC-MS, HPLC, HPLC/UV, UPLC-MS/MS, GC–MS, DLLME-GC–MS, and ELISA, as summarized in Table 2. Among these, GC–MS, LC-MS, and their hyphenated variants are widely employed due to their broad applicability and generally superior sensitivity, often achieving detection limits in the low parts-per-billion (ppb) range.

**Table 2:** Detection methods for AA, Furan, AGEs and MI in baked foods.

**Analytical method**	**Sample**	**TPHs**	**LOD**	**LOQ**	**Recovery**	**Reference**
LC-MS/MS	bread crumb	AA		10 μg/kg		(Bartkiene et al., 2013)
	bread	AA		5 μg/kg		(Hedegaard, Granby, Frandsen, Thygesen, & Skibsted, 2008)
	ammonia	AA	0.2 ng/mL	0.8 ng/mL		(Mousa, 2019)
	biscuits	AA	0.05 mg/kg			(Nguyen et al., 2016)
	cookies	AA	10 ng/mL			(Fiore et al., 2012)
	bread toasted	MI		10 μg/kg	85%	(Mottier, Mujahid, Tarres, Bessaire, & Stadler, 2017)
	bread	MI		20 μg/kg	91–113%	(Wu, Wang, Li, & Yu, 2019)
LC-MS	cookies	AA		15 ng/mL		(Açar & Gökmen, 2010)
HPLC	shortbread	AA	8 μg/kg	28 μg/kg	90.5–99.1%	(Mousa, 2019)
	bread	AGEs	42 μg/kg	1.29 μg/kg	100%	(Mildner-Szkudlarz et al., 2017)
HPLC/UV	bread	AA	25 μg/kg	80 μg/kg	90.6–109.8%	(Albishri & Abd El-Hady, 2014)
UPLC-MS/MS	cookies	AA	0.024 μg/mL	0.077 μg/mL	88.24%	(Xue et al., 2022)
	whole meal bread	AGEs	0.024 μg/mL	0.2 mg/kg		(Scheijen et al., 2016)
	whole meal bread	AGEs	0.5 mg/kg	1.6 mg/kg		(Hull, Woodside, Ames, & Cuskelly, 2012)
	biscuit	MI		5 μg/kg	76–112%	(Jacobs et al., 2018)
	cookies	MI	0.014 μg/mL	0.021 μg/kg	88.24%	(Xue et al., 2022)
	bread	MI		5 μg/kg		(Jacobs et al., 2018)
GC–MS	bread	AA	7.46 μg/kg	24.88 μg/kg	83%	(Boyacı Gündüz & Cengiz, 2015)
	bakery	AA	25 μg/kg	75 μg/kg	82.9–104.25%	(Pacetti et al., 2015)
	ammonia biscuits	MI	19 ng/mL	53 ng/mL		(Mousa, 2019)
DLLEGC–MS	cake	AA	0.6 μg/kg	2 μg/kg	95%	(Nematollahi et al., 2019)
ELISA	cracker	AA	85.0 ± 4.2 μg/L		90%–110.5%	(Sun, Xu, Ma, Qiao, & Xu, 2014)
	cakes	AGEs				(Takeuchi et al., 2015)
HS-Spme-GC/FID	fruit juices	Furan	0.14–0.76 μg/L	0.056–0.230 μg/L		(Hu, Zhu, Hernandez, Koutchma, & Shao, 2016)
HS-Spme-GC/MS	orange juices	Furan	0.26 μg/L	0.079 μg/L		(Kim, Kim, & Lee, 2016)
HS-GC–MS/MS	baby food	Furan	0.071 μg/kg	0.021 μg/kg		(Pugajeva, Rozentale, Viksna, Bartkiene, & Bartkevics, 2016)
HS-GC/MS	food samples	Furan	0.5–10.0 μg/kg	0.2–5.0 μg/kg		(Shen et al., 2016)
HS-Trap-GC/MS	bread	Furan	0.1 μg/kg	0.03 μg/kg		(Huault et al., 2016)
HS-Spme-Arrow-GC-MS/MS	food samples	Furan	0.003–3.571 μg/kg	0.001–1.071 μg/kg		(Huang, Kao, Inbaraj, & Chen, 2022)
LC-HRMS	bread	MI	0.5 μg/kg	1 μg/kg	85.4–93.7%	(Chen, Yan, Lv, Zhao, & Wu, 2018)

#### 3.1.2 Control methods and techniques for *AA*

##### 3.1.2.1 Control of precursor substances

Based on the formation mechanism of *AA*, reducing the concentrations of key substrates, namely asparagine and reducing sugars, can effectively lower *AA* levels in bakery products (Sazesh & Goli, 2020). Since most bakery products are derived from cereals, controlling the content of *AA* precursors at the crop level through careful selection of cereal varieties and optimized cultivation conditions can mitigate *AA* formation. Specifically, choosing cereal varieties with naturally low asparagine content has been shown to reduce *AA* in cereal-based products (). Additionally, enzymatic treatment with asparaginase prior to processing can hydrolyze asparagine into aspartic acid, resulting in a 34%–94% reduction in asparagine content compared to untreated samples. Importantly, asparaginase exhibits high specificity for asparagine without affecting the levels of other amino acids (Peivasteh-Roudsari et al., 2024; Sarion, Codină, & Dabija, 2021).

##### 3.1.2.2 Control of processing conditions

*AA* content is significantly correlated with pH, baking time, and in bakery products, the addition of organic acids can be used to precisely modulate the initial system pH. Under acidic conditions (pH < 6.0), asparagine undergoes hydrolysis of its carboxamide group, reducing the availability of free asparagine for the Maillard reaction that drives *AA* formation, thereby lowering *AA* content (Zhang et al., 2012). Baking parameters also play a critical role in *AA* formation, particularly in the crust, where over 99% of *AA* is produced. Capuano, Ferrigno, Acampa, Ait-Ameur, and Fogliano (2008) demonstrated that *AA* formation in the crust is strongly dependent on baking time and temperature, with temperatures exceeding 200 °C substantially increasing *AA* levels.

##### 3.1.2.3 Addition of exogenous additives

The addition of exogenous additives represents a promising strategy for mitigating the formation of hazardous compounds in bakery products. Non-aspartic amino acids, such as glycine, lysine, and cysteine, have been shown to effectively reduce *AA* levels; for example, Zou et al. (2015) demonstrated that adding 0.36% cysteine and 0.2% glycine to cookies achieved a 97.8% reduction in *AA* content. In addition to amino acids, protein analogs such as amaranth protein can also inhibit amino acid formation. Salazar, Arámbula-Villa, Vázquez-Landaverde, Hidalgo, and Zamora (2012) reported inhibition rates of 35–40% in simulated systems and 89% in crackers. Similarly, olive polyphenols studied by Troise, Colantuono, and Fiore (2020) showed the greatest inhibition of *AA* formation, reaching 55% at a 0.1% addition to crackers. Furthermore, Morales et al. (2014) also reported inhibition rates of 35–40% in simulated systems and 89% in crackers. (2014) observed that natural extracts (e.g., oregano, thyme, cinnamon, bougainvillea, and green tea) with high free radical inhibition rates (48–99%) and phenolic contents (205–547 mg GAE/g dry weight) reduced the *AA* content of fried potatoes by up to 62% (green tea), 39% (cinnamon), and 17% (oregano) after soaking for 1 min before frying. These findings highlight the potential of exogenous additives, particularly polyphenols, to inhibit *AA* formation during thermal processing. However, the practical application of such additives requires careful consideration of potential drawbacks. These may include altered sensory profiles (e.g., undesirable flavor or color imparted by amino acids or polyphenols), increased production costs (particularly for highly purified or natural extracts), and the need to ensure compliance with relevant food safety regulations regarding the levels and types of additives used.

##### 3.1.2.4 Fermentation process

Fermentation employing lactic acid bacteria (LAB) or yeast represents a promising strategy for reducing *AA* levels in bakery products. Albedwawi et al. (2021) demonstrated this effectively by incorporating yeast alongside strains of *Lactobacillus plantarum* and *Lactobacillus brevis* during bread production, achieving an 84.7% reduction in *AA* formation. Furthermore, co-fermentation strategies utilizing both LAB and yeast have been investigated for *AA* mitigation. Studies confirm significantly lower *AA* content in bread produced via LAB-yeast co-fermentation (6.9–20 μg/kg) compared to bread fermented solely with yeast (47.6 μg/kg).

##### 3.1.2.5 New food processing technology

Traditional baking processes are characterized by long internal baking times and relatively low thermal conductivity. This often results in low surface water activity and high heat input, factors that contribute to elevated amino acid levels. New processing technologies can effectively mitigate this issue: Vacuum baking: Vacuum technology significantly reduces amino acid formation during baking by lowering the boiling point and shortening the processing time. Lee et al. (2019) demonstrated its potential, while Mogol and Gökmen (2014) demonstrated that amino acid concentrations in vacuum-baked cookies were significantly lower than those in conventionally baked cookies at various temperatures (160 °C, 180 °C, and 200 °C). However, complete amino acid removal at very low pressures is difficult due to matrix viscosity, which hinders molecular diffusion. Hydration pretreatment before vacuum application can alleviate these process challenges, particularly for baked products such as cookies, fruitcakes and whole-wheat flatbreads. Relying solely on vacuum often hinders the Maillard reaction, resulting in insufficient surface browning and suboptimal flavor development. A hybrid approach addresses this limitation: an initial high-temperature heat treatment (220 °C for 2–4 min) triggers Maillard browning and flavor development; subsequent vacuum-assisted accelerated drying (180 °C, 500 mbar for 4–6 min) effectively reduces heat exposure time. As demonstrated by Mogol and Gökmen (2014), this staged strategy suppresses harmful processing hazards such as *AA* while maintaining structural integrity and mitigating the sensory deficiencies inherent in pure vacuum baking. Both vacuum baking and hot-vacuum hybrid approaches represent promising technological advances, significantly reducing *AA* levels in baked products compared to traditional methods while aiming to maintain acceptable sensory and textural qualities. However, to fully realize their industrial potential and promote their wider application, future research needs to address key challenges. Key points include overcoming the high cost of specialized vacuum equipment; optimizing process parameters (such as pressure, temperature, and water activity) for different product matrices to ensure consistent efficacy and quality; and designing precise control strategies to inhibit the production of harmful substances such as *AA* while maximizing the desired color, aroma, and taste. In addition, interdisciplinary research that integrates disciplines such as food materials science is crucial. This not only promotes the innovation of scalable solutions, but also deepens the understanding of the complex physical and chemical changes in these new processing processes. Solving the above challenges is key to translating these promising technologies into industrial applications that meet strict safety standards and do not affect consumer acceptance.

### 3.2 Detection method and control method and technology for *5-HMF*

#### 3.2.1 Detection method for *5-HMF*

According to the available reports, the most widely used technique for the detection of *5-HMF* is the HPLC technique, the researchers selected three samples such as ammonia biscuit, cookies and croissants respectively, and the results of LOD and LOQ for ammonia biscuit were 0.01 ng/mL and 0.09 ng/mL, respectively (Mousa, 2019), LOD for cookies was 1.0 mg/kg (Huault et al., 2016), and LOD and LOQ for croissants were 0.050 mg/kg and 0.150 μg/mL for croissants (Fiore et al., 2012).

#### 3.2.2 Control technology for *5-HMF*

##### 3.2.2.1 Control of precursor substances

Free amino acids in food have an important influence on the formation of *5-HMF*. Capuano et al. (2009) found in their experiments that the use of refined flour, which is lower in free amino acids, produced less *5-HMF* during processing than plain flour. It was found that the addition of a certain amount of sodium bicarbonate during cookie production with glucose and sucrose as raw materials was found to reduce *5-HMF* levels, likely because sodium bicarbonate maintains alkalinity (pH ≈ 9.0) during baking, limiting the hydrolysis of sucrose into reducing sugars that serve as *5-HMF* precursors (Gökmen, Açar, Serpen, & Morales, 2008). It has been concluded that the *5-HMF* content of foods with high sugar content rises during thermal processing (Zhang et al., 2012). Therefore, by carefully controlling sucrose addition and processing at lower temperatures, it is theoretically possible to produce bakery products with reduced *5-HMF* content.

##### 3.2.2.2 Control of processing conditions

The *5-HMF* content increases with baking time (Hu et al., 2022). Researchers found that increasing the moisture content in the sample during processing also affected its *5-HMF* concentration. Increasing the moisture content from 22% to 26% significantly reduced the *5-HMF* content by 43% (Fredriksson, Tallving, Rosen, & Åman, 2004).

##### 3.2.2.3 Addition of exogenous additives

Zhang and An (2017) observed the changes in *5-HMF* content by adding quercetin to wheat bread and found that quercetin inhibited the formation of *5-HMF* and its precursors to different degrees. Researchers found that baking at 160 °C for 30 min maximized quercetin's inhibitory effect on *5-HMF* formation (86.0%). This indicates that controlling temperature and time, alongside adding exogenous additives, can enhance inhibition. Similarly, adding cysteine and glycine, alone or combined, markedly reduced *5-HMF* in cookies, with 0.36% cysteine and 0.2% glycine lowering levels by 93.2% (Zou et al., 2015). Although salts generally promote *5-HMF* formation, NaCl encapsulation was shown to inhibit it by raising the temperature required for sucrose hydrolysis, thereby limiting fructose and *5-HMF* accumulation without affecting cookie quality (Fiore et al., 2012). Similarly, adding hydrophilic colloids such as gum arabic reduced *5-HMF*, with 0.05 g achieving 74% inhibition after baking at 180 °C for 10 min (Mousa, 2019).

In addition, researchers have suggested that low-calorie processing methods, such as microwave heating, can significantly reduce *5-HMF* formation in bakery products as part of novel food processing technologies.

### 3.3 *Furan* detection methods and control methods and technologies

#### 3.3.1 Methods for the detection of *furan*

According to available reports, the most commonly used techniques for *furan* detection include HS-SPME-GC/FID, HS-SPME-GC/MS, HS-GC–MS/MS, HS-GC/MS, HS-Trap-GC/MS, and HS-SPME-Arrow-GC–MS/MS, as summarized in Table 2.

#### 3.3.2 Control technology for *furan*

*Furan* control strategies must balance the reduction of *furan* content with the preservation of desirable organoleptic and nutritional properties. Reported approaches can be categorized into four main areas: (a) removal or substitution of food components to control *furan* precursors, (b) addition of exogenous additives, particularly antioxidants, (c) modification of processing conditions, and (d) application of novel food processing technologies (Fardella et al., 2021; Kettlitz et al., 2019).

##### 3.3.2.1 Control of precursor substances

Guo et al. (2019) reported that using xylitol as a complete substitute for *furan* precursors in systems containing sucrose and high-fructose corn syrup achieved inhibition rates of up to 88.6%. This finding suggests that directly targeting precursor substances can be a more efficient and cost-effective strategy for *furan* control compared to relying on large instrumentation or more complex methods, although each approach presents its own advantages and limitations.

##### 3.3.2.2 Changing processing conditions

Sevenich et al. (2014) reported that autoclave sterilization during processing reduced *furan* concentrations by 81%–96% compared to conventional distillation methods. Additionally, high hydrostatic pressure treatment has proven highly effective, as Kultur et al. (2018) detected no *furan* in samples following such processing.

##### 3.3.2.3 Addition of exogenous additives

Exogenous additives, particularly antioxidants and polyphenols, have been widely employed to reduce *furan* content in bakery products. Zheng, Chung, and Kim (2015) reported significant decreases in *furan* levels by adding catechins (44.7%), ferulic acid (57.6%), α-tocopherol (39.3%), β-carotene (34.8%), sodium sulfite (64.1%), and glutathione (44.9%). Similarly, Bi et al. (2017) observed up to 42.4% inhibition of *furan* formation upon adding 84 mg of tea polyphenols to the system. Furthermore, Wang et al. (2021) demonstrated that anthocyanin extracts from red apples reduced *furan* content by 20% in coffee powder and 68% in NFC apple juice, highlighting the effectiveness of polyphenolic compounds as *furan* inhibitors.

##### 3.3.2.4 New food processing technology

Hradecky, Kludska, Belkova, Wagner, and Hajslova (2017) reported that ohmic heating, a novel food processing technique, reduced *furan* content by 70%–90% compared to conventional retort processing. Vacuum treatment has also been widely applied as an effective processing strategy: Anese et al. (2014) achieved up to 67% *furan* removal in meat sauce under conditions of 12 kPa for 10 min, while Palmers et al. (2016) observed up to 60% *furan* reduction in various potato models using vacuum treatment.

### 3.4 Detection method and control technology for *AGEs*

#### 3.4.1 Techniques for the detection of *AGEs*

According to the available reports, the predominant analytical techniques for detecting advanced *AGEs* include HPLC, UPLC-MS/MS, and ELISA, as summarized in Table 2. Among these, UPLC-MS/MS has garnered significantly wider adoption than alternative techniques. This preference primarily stems from its superior analytical capabilities: 1) UPLC-MS/MS has a relatively lower limits of detection (LOD) and quantification (LOQ), enabling the reliable measurement of trace-level *AGEs* in complex matrices like bakery products. 2) The tandem mass spectrometry (MS/MS) stage provides high selectivity through precursor ion selection and specific fragment ion monitoring, effectively distinguishing target *AGEs* from co-eluting matrix interferences, thereby reducing false positives and negatives. 3) Compared with traditional HPLC, UPLC has higher chromatographic resolution and faster separation time. ELISA and HPLC are limited to detecting only some *AGEs*, UPLC-MS/MS can simultaneously detect, identify, and quantify a wide range of individual AGE compounds in a single analysis.

#### 3.4.2 Techniques for the control of *AGEs*

##### 3.4.2.1 Control of precursor substances

When making baked products, try to choose raw materials with low reducing sugar content, as studies have shown that glucose and fructose, two reducing sugars, will produce relatively high concentrations of carboxymethyllysine and carboxyethyllysine after baking (Mildner-Szkudlarz, Siger, Szwengiel, & Bajerska, 2015).

##### 3.4.2.2 Changing processing conditions.

Processing temperature significantly influences the formation of *AGEs* in baked goods. Notably, CML, a prominent AGE, exhibits a temperature-dependent profile characterized by a formation-degradation kinetic pattern. In biscuit production, CML formation dominates at temperatures up to 200 °C, driven primarily by the Maillard reaction and its associated sugar oxidation pathway, resulting in peak CML accumulation. However, at significantly higher temperatures (e.g., 300 °C), thermal degradation processes become more pronounced. The CML molecules themselves, as well as key precursors and intermediates formed early during heating, may undergo degradation or destructive reactions (e.g., pyrolysis, oxidation). As a result, the measured CML content in the final product decreases at 300 °C compared to 200 °C. This shift from net formation to net degradation at elevated temperatures has been quantitatively confirmed through chromatographic analysis of baked cookies (Žilić, Aktağ, Dodig, & Gökmen, 2021).

##### 3.4.2.3 Addition of exogenous additives

The incorporation of polyphenolic compounds (e.g., quercetin, anthocyanins) and flavonoids during baking offers an effective strategy for mitigating *AGEs* formation. These additives act primarily through two mechanisms: (1) scavenging reactive free radicals to inhibit oxidative reactions, and (2) directly trapping α-dicarbonyl intermediates, such as glyoxal and methylglyoxal, via covalent adduct formation, thereby preventing their reaction with amino groups to form *AGEs*. Peng et al. (2010) reported that the addition of grape seed extract (GSE), rich in phenolic acids, flavonoids, and proanthocyanidins, significantly reduced CML levels in bread crust, with suppression exceeding 50% at GSE concentrations of 1200 mg/kg and 2000 mg/kg, attributed to its potent free radical scavenging capacity inhibiting α-dicarbonyl precursor formation. Similarly, Teng et al. (2018) demonstrated that adding dihydromyricetin effectively suppressed *AGEs* formation in a cookie model system, consistent with the established radical scavenging and dicarbonyl trapping capabilities of polyphenols and flavonoids.

### 3.5 Detection methods and control technologies for *HCAs*

#### 3.5.1 Detection of *HCAs*

*HCAs*, food-borne carcinogens formed during thermal processing, have been widely studied in the past decade (Özdestan, Kaçar, Keşkekoğlu, & Üren, 2014). Their quantification is difficult due to ultra-low levels in complex food matrices (0.1–50 ng/g), requiring extensive purification methods such as liquid-liquid extraction, SPE, blue cotton extraction, or immunoaffinity chromatography. Detection commonly exploits their structural features: many *HCAs* show strong intrinsic fluorescence from fused heteroaromatic rings, and all exhibit distinct UV absorption from conjugated π-electron systems. High-performance liquid chromatography with fluorescence detection (HPLC-FLD) offers high sensitivity and selectivity for naturally fluorescent *HCAs*, while HPLC-UV applies more broadly. LC-MS/MS enables precise identification and quantification at trace levels, and ELISA and pressurized liquid extraction (PLE) also support HCA analysis.

#### 3.5.2 Methods and techniques for the control of *HCAs*

Control strategies for *HCAs* in thermally processed foods primarily fall into three categories: First, process modification involves altering processing methods or conditions, such as reducing temperature, optimizing cooking time, or increasing surface moisture and water activity, thereby directly targeting the key drivers of *HCAs* formation, which typically occur at temperatures above 150 °C under low-moisture conditions (Zeng et al., 2018). Second, additive incorporation utilizes antioxidants and plant extracts to suppress *HCAs* formation through specific biochemical pathways. Water-soluble vitamins and polyphenols, including ascorbic acid (vitamin C), pyridoxine, thiamine, pyridoxamine, biotin, and nicotinic acid, have demonstrated more than 40% inhibition in model systems (Wong, Cheng, & Wang, 2012). Ascorbic acid functions primarily by scavenging free radicals, interrupting radical-mediated reactions crucial for *HCAs* synthesis, while pyridoxine sequesters α-dicarbonyl intermediates like phenylglyoxal, blocking downstream formation pathways. Antioxidant-rich plant extracts, such as hawthorn and pomegranate seed, also inhibit *HCAs*, though their use requires careful management of sensory effects (bitterness, astringency, or color changes) and adherence to regulatory safety limits. Third, novel processing techniques, such as microwave pre-treatment, modify the physicochemical properties of key HCA precursors, including creatine, creatinine, and free amino acids, by accelerating hydrolysis and promoting leaching into expelled water, thereby reducing precursor availability for Maillard reactions during subsequent high-temperature cooking (frying or grilling). Studies have reported up to a 30% reduction in precursor compounds, resulting in a substantial 95% decrease in the mutagenic activity of formed *HCAs*.

### 3.6 Methods and techniques for the detection and control of *MI*

#### 3.6.1 Detection of *MI*

According to available reports, the predominant analytical techniques for detecting *MI*, notably *2-MI* and *4-MI*, in food matrices include LC-MS/MS, liquid chromatography-high-resolution mass spectrometry in parallel reaction monitoring mode (LC-HRMS/PRM), UPLC-MS/MS, and GC–MS, as summarized in Table 2. Among these, UPLC-MS/MS has seen the widest adoption, largely due to its superior analytical performance for trace-level quantification, which is critical for *MI* typically present at ng/g concentrations. This technique combines enhanced chromatographic resolution, achieved using sub-2 μm particle columns under high-pressure conditions, with the high sensitivity and selectivity of MS/MS detection in multiple reaction monitoring (MRM) mode, ensuring precise and accurate quantification at very low concentrations. Consequently, UPLC-MS/MS has become the benchmark method for the reliable analysis of *2-MI* and *4-MI* in complex food matrices.

#### 3.6.2 Methods and techniques for the control of imidazole compounds

##### 3.6.2.1 Changing processing conditions

It has been shown that the concentration of *4-MI* gradually increases with rising baking temperature and extended thermal exposure. Xue et al. (2022) showed that the *4-MI* concentration reached a maximum after baking the sample at 200 °C for 11 min. Since the formation pathway of *2-MI* is different from that of *4-MI*, *2-MI* does not increase in concentration with increasing baking temperature and time.

##### 3.6.2.2 Addition of exogenous additives

The addition of food additives and polyphenols during baking can reduce the concentration of imidazole compounds to a certain extent. Among the additives, researchers are currently focusing more on caramel colorants (Jung, Kim, & Lee, 2017). In biscuit samples, the addition of 0.2% and 1.0% caramel colorants and baking at 140 °C for 8 min reduced the *4-MI* content by a maximum of 56% and 34%, respectively (Xue et al., 2022). Adding curcumin to a biscuit simulation system significantly inhibited the formation of methylglyoxal by scavenging free radicals and limiting the utilization of precursors, reducing its concentration by a maximum of 51.55%. Adding gum arabic and pectin to biscuit dough also inhibited the formation of 4-methylimidazolaldehyde (Mousa, 2019). Among them, gum arabic can significantly reduce the formation of *4-MI* after baking at 180 °C for 10 min. Adding 0.05 g of gum arabic can inhibit 89.9% of *4-MI*, which has a good inhibitory effect and is a very promising control method. However, for this type of polyphenols, the specific properties of the colloid need to be further studied.

### 3.7 Synergies between multiple approaches to hazard control

While single hazard control approaches have demonstrated effectiveness, emerging reports indicate that synergistic mitigation can be achieved through the combination of multiple technologies, which is more powerful and comprehensive than any single approach. This synergy primarily stems from multi-target intervention strategies, where different technologies simultaneously address different pathways or risk factors, thereby overcoming the inherent limitations of a single approach. Several examples illustrate this principle: Xue et al. (2023) showed that combining curcumin addition (through free radical scavenging and dicarbonyl capture) with microwave pretreatment reduced *AA* formation in biscuits by 92%, significantly exceeding the effect of either method alone (curcumin: 51%, microwave: 68%). Similarly, combining yeast fermentation (consuming the precursor asparagine and lowering pH) with vacuum baking (reducing reaction temperature and removing volatile substances) more effectively preserved product flavor while inhibiting *AA* and *5-HMF* formation. Zhang et al. (2020) also reported that the combined use of calcium chloride (chelating agent), L-asparaginase (enzyme precursor reducer), and moderate infrared heating achieved superior *AA* control in potato chips compared to any single or dual intervention, highlighting the synergistic benefits of chemical, enzymatic, and physical strategies. Further, the synergistic application of specific amino acids (e.g., lysine and glycine) as alternative nucleophiles or carbonyl traps, together with optimized pulsed electric field (PEF) pretreatment, significantly reduced *furan*s and *AGEs* by interfering with multiple stage*s* of the Maillard reaction cascade. Beyond thermal hazards, the combination of mild ultrasound (which enhances mass transfer and breaks down cell walls) with natural antimicrobials (such as nisin and plant extracts) has been shown to have synergistic effects against spoilage microorganisms and pathogens in fillings or doughs, thereby reducing the intensity of each treatment and improving quality retention. However, implementing such synergistic control strategies requires careful consideration of potential trade-offs: sensory impairments such as bitterness or astringency from added polyphenols or unpleasant color (e.g., intense yellow color from high-dose curcumin) may occur, while non-thermal techniques such as microwave heating can sometimes lead to uneven browning or variations in crust formation. Process complexity and cost are also issues, as integrating multiple technologies (e.g., fermentation + vacuum baking + additive systems) increases equipment investment, operational complexity, energy consumption, and validation requirements; purchasing high-purity natural additives further increases raw material costs. Furthermore, regulatory hurdles must be anticipated, as new process-additive combinations often require extensive safety assessments and face intense scrutiny. Therefore, future research should prioritize optimizing synergistic combinations. As such, it should 1) identify the most effective multi-target intervention measures aimed at specific hazards (e.g., mitigating *AGEs* via antioxidants and pH regulators), and 2) determine optimal additive types and dosages to minimize sensory impacts while ensuring product safety and quality. Mastering these multi-faceted methods is crucial for achieving comprehensive control of harmful substances in hot-processed foods.

## 4 Conclusions

The hazards caused by thermal processing of food raw materials were worthy of attention, especially *AA*, *5-HMF*, *furan*, *AGEs*, *HCAs* and *imidazole*. The formation mechanisms of these compounds are often closely linked to the Maillard reaction, caramelization, and lipid oxidation. Currently, their detection primarily relies on advanced analytical techniques such as gas chromatography (GC), liquid chromatography (LC) and their mass spectrometry-coupled variants (GC–MS, LC-MS), which enable sensitive and precise quantification even at trace levels. Control strategies for these hazards mainly focus on two approaches: first, the addition of relevant exogenous substances to inhibit their formation, and second, optimization of processing operations and conditions to limit hazard generation during thermal treatment. At present, it is the key challenge to effectively reduce the harm, control the industrial scalability and compliance of laws and regulations without affecting the sensory quality of baked foods.

## 5 Future challenges and research needs

(1) The formation mechanisms of thermal processing hazards require further in-depth investigation. Currently, research on harmful substances in thermally processed foods has primarily focused on *AA*, *furan* and *AGEs*, while issues related to *5-HMF* remain underexplored and warrant more detailed study.
(2) Advanced and intelligent detection methodologies. The integration of machine learning with spectroscopic and sensor technologies presents a transformative direction for future hazard analysis. By processing complex spectral data from techniques like hyperspectral imaging or Raman spectroscopy, machine learning models can significantly enhance detection throughput, accuracy, and the capability for simultaneous identification of multiple co-existing hazards in baked products. This data-driven approach is key to developing rapid, non-destructive, and potentially portable tools for in-line monitoring, moving beyond reliance on central laboratory analysis.
(3) Optimization of thermal processing parameters: Emerging technologies, such as microwave-assisted heating, infrared processing, and ohmic heating, show promise for mitigating thermal processing hazards. However, their industrial application remains limited by challenges in preserving the texture and flavor profiles characteristic of conventional baking. Effectively overcoming these limitations will require close multidisciplinary collaboration among food chemists, process engineers, and sensory scientists.
(4) Development of multi-hurdle intervention strategies. A promising approach involves integrating existing mitigation strategies, modification of precursor substrates, and optimization of time-temperature protocols, with novel physical field-assisted processing technologies. This synergistic methodology enables a comprehensive evaluation and optimization, which can be systematically guided using response surface methodology.

In the context of frequent food safety incidents, it is essential to effectively respond to these challenges for guiding food production, improving quality consistency and safety, and finally addressing the urgent global concerns surrounding food safety.

## CRediT authorship contribution statement

**Qi Zhang:** Investigation, Formal analysis. **Pei Liu:** Writing – review & editing, Supervision, Resources, Methodology. **Yanhong Ma:** Writing – review & editing, Visualization, Software, Methodology, Investigation, Data curation, Conceptualization. **Yuhua Diao:** Writing – review & editing, Validation. **Ying Gu:** Writing – review & editing, Supervision, Funding acquisition. **Xuejing Fan:** Writing – review & editing, Validation, Formal analysis.

## Declaration of competing interest

The authors declare that they have no known competing financial interests or personal relationships that could have appeared to influence the work reported in this paper.

## Data Availability

Data will be made available on request.
